# Phylogenetic evidence of a novel lineage of canine pneumovirus and a naturally recombinant strain isolated from dogs with respiratory illness in Thailand

**DOI:** 10.1186/s12917-019-2035-1

**Published:** 2019-08-19

**Authors:** Chutchai Piewbang, Somporn Techangamsuwan

**Affiliations:** 1 Department of Pathology, Faculty of Veterinary Science, Chulalongkorn University, Bangkok, 10330 Thailand; 2 Diagnosis and Monitoring of Animal Pathogens Research Unit, Faculty of Veterinary Science, Chulalongkorn University, Bangkok, 10330 Thailand

**Keywords:** Canine pneumovirus, Genetic recombination, Phylogenetic analysis, Thailand

## Abstract

**Background:**

Canine pneumovirus (CPV) is a pathogen that causes respiratory disease in dogs, and recent outbreaks in shelters in America and Europe have been reported. However, based on published data and documents, the identification of CPV and its variant in clinically symptomatic individual dogs in Thailand through Asia is limited. Therefore, the aims of this study were to determine the emergence of CPV and to consequently establish the genetic characterization and phylogenetic analysis of the CPV strains from 209 dogs showing respiratory distress in Thailand.

**Results:**

This study identified and described the full-length CPV genome from three strains, designated herein as CPV_CP13 TH/2015, CPV_CP82 TH/2016 and CPV_SR1 TH/2016, that were isolated from six dogs out of 209 dogs (2.9%) with respiratory illness in Thailand. Phylogenetic analysis suggested that these three Thai CPV strains (CPV TH strains) belong to the CPV subgroup A and form a novel lineage; proposed as the Asian prototype. Specific mutations in the deduced amino acids of these CPV TH strains were found in the G/glycoprotein sequence, suggesting potential substitution sites for subtype classification. Results of intragenic recombination analysis revealed that CPV_CP82 TH/2016 is a recombinant strain, where the recombination event occurred in the L gene with the Italian prototype CPV Bari/100–12 as the putative major parent. Selective pressure analysis demonstrated that the majority of the nucleotides in the G/glycoprotein were under purifying selection with evidence of positive selection sites.

**Conclusions:**

This collective information on the CPV TH strains is the first evidence of CPV emergence with genetic characterization in Thailand and as first report in Asia, where homologous recombination acts as a potential force driving the genetic diversity and shaping the evolution of canine pneumovirus.

**Electronic supplementary material:**

The online version of this article (10.1186/s12917-019-2035-1) contains supplementary material, which is available to authorized users.

## Background

Canine pneumovirus (CPV) was first identified in 2010 from American dogs that showed respiratory disease [1] and has recently been considered as one of the etiological agents of canine infectious respiratory disease complex (CIRDC) [2–6]. Initially, the CnPnV was abbreviately named for the canine pneumovirus; however, to make species names uniforms, the CnPnV was recently replaced by “CPV” acronym, which is established by the International Committee on Taxonomy of Viruses (ICTV) [7]. CPV belongs to the *Pneumoviridae* family, genus *Orthopneumovirus*, which includes viruses associated with both animal and human respiratory pathogens, such as human respiratory syncytial viruses (HRSV), bovine respiratory syncytial virus (BRSV), murine pneumonia virus (MPV) and the recently discovered novel swine orthopneumovirus (SOV) [8]. Complete genome sequence analysis of the previously described CPV strains indicated that it was very highly related to the MPV with 95–96% nucleotide identity [1, 2, 9]. However, CPV has been taxonomically segregated as an unclassified virus as well as caprine pneumovirus and ovine pneumovirus [7].

As a *Pneumoviridae*, CPV contains six core genes, which are arranged as NS1-NS2-N-P-M-SH-G-F-M2-L [2, 10]. The non-structural (NS) proteins, NS1 and NS2, are highly unique among the pneumoviruses and are associated with pathogenicity and alteration of interferon responses in MPV and HRSV infections [11, 12]. The phosphoprotein (P) and large (L) protein are essential to form the core RNA-dependent RNA polymerase complex with the N protein to make up the complete nucleocapsid [10]. The matrix (M) protein is a structural protein that enhances the association of the nucleocapsid with its membrane. For the MPV, the M2 gene acts as transcriptional regulation for RNA replication [13]. The major surface glycoproteins contain the small hydrophobic (SH) protein, attachment glycoprotein (G/glycoprotein) and the fusion (F) protein that both are essential to the pneumovirus infection [14, 15]. The G/glycoprotein targets the ciliated cells in the trachea while the F protein enhances the viral membrane fusion with the membrane of target cells for RSVs infection [15]. Furthermore, both the G/glycoprotein and F proteins are targets for neutralizing antibodies following infection of HRSV [16, 17] and BRSV [18]. Together, the G/glycoprotein and F glycoproteins are considered an important virulence factor, and have a high level of genetic and antigenic variation between related pneumovirus species, which frequently gives rise to escape mutants [19–23]. Therefore, both the G/glycoprotein and F genes serve as target genes for the analysis of genetic diversity of these viruses. Indeed, a previous study that focused on CPV isolation from dogs and cats in the USA attempted to define the CPV subgroup on the basis of a molecular study of both the G/glycoprotein and F genes [9].

Since CPV was discovered in 2010, investigations into the prevalence of CPV in respiratory illness dogs have been conducted and reported as outbreaks in various canine breeding colonies and shelters in the USA [9, 10] and various countries in Europe [2, 5, 6]. Furthermore, retrospective surveys of CPV seroprevalence in European dogs revealed an estimated CPV-seropositive level of about 50% in pet dogs and a markedly higher level of up to 93.5% in kenneled dogs [3, 6]. Thus, CPV has likely spread over various areas in America and Europe and is not geographically limited. However, there is no information regarding the emergence of CPV in Asian countries.

Recombination plays a crucial role in the evolution of RNA viruses [24]. Recent evidence of natural recombination forcing the evolution of pneumoviruses has been documented in HRSV [25, 26], human metapneumovirus [27] and avian metapneumovirus [28], but evidence of natural genetic recombination in the CPV genome is not yet available. To complete this fragmentary data, this study provided molecular identification and characterization of the full-length genome of three Thai CPV (CPV TH) strains isolated from the airways of dogs with respiratory disease in Thailand. The determination and analysis of isolated CPVs led us to define the first identification of CPV Asian strains, which were phylogenetically distinct from the known American and European strains. Beyond this finding, we have analyzed the potential recombination events in the Asian CPVs and found that one of the three Asian CPV strains was a recombinant virus. Therefore, the information presented in this study is essential for studying the CPV diversity and evolution and gives evidence for the first emergence of CPV in Asia.

## Methods

### Ethics and consents

All experimental protocols were approved by the Chulalongkorn University Animal Care and Use Committee (No. 1631002). All procedures were done in accordance with the relevant guidelines and regulations. The dog owners gave his/her written consent for sample collection and data publication. The collected samples were carried and managed by an approval of Institutional Biosafety Committee (IBC No. 1631002) in accordance with the regulations and policies governing the Biosafety.

### Animals and clinical specimens

A total of 418 respiratory specimens (equally nasal and oropharyngeal swabs) were obtained from 209 dogs showing respiratory distress. The samples were collected from client-owned dogs from various areas in Thailand during 2014–2016. General signalment was noted during the time of sampling, including the age, sex, breed, vaccination status and related respiratory clinical signs. The swabs were immersed in 500 μL of 1% sterile phosphate buffered saline and submitted by using triple pack system to Department of Pathology, Faculty of Veterinary Science, Chulalongkorn University and then kept at − 80 °C until used.

### Nucleic acid extraction

Total nucleic acids were extracted from 200 μL of collected samples using the Viral Nucleic Acid Extraction Kit II (GeneAid, Taipei, Taiwan) according to the manufacturer’s recommendation. The extracted nucleic acid was then quantified and qualified by spectrophotometric analysis with a Nanodrop® Lite (Thermo Fisher Scientific Inc., Waltham, MA, U.S.A.). The nucleic acids were then kept at − 80 °C until assayed.

### Respiratory viral screening

Extracted nucleic acids were subjected to routine laboratory viral investigation. Common viruses associated with canine infectious respiratory disease complex (CIRDC) comprised of canine influenza virus, canine parainfluenza virus, canine distemper virus, canine respiratory coronavirus, canine adenovirus type 1 and 2 and canine herpesvirus type 1, were screened by multiplex PCRs as described previously [29]. Furthermore, one-step Pan-RT-PCR using a specific primer set for broad-ranged paramyxo-pneumoviruses (PMX) was then performed as described previously with some minor modifications [30].

Subsequently, PCR products were run on the QIAxcel capillary electrophoresis (QCES) platform. Briefly, the PCR-amplified fragments were analyzed based on high throughput capillary electrophoresis using QIAxcel DNA High Resolution Kit (Qiagen, Hilden, Germany). A custom alignment marker of 15–1000 bp was simultaneously run with the samples. The QIAxcel DNA size marker of 50–800 bp was used for size estimation. The samples were analyzed using the default OM500 method at 5 kV of separation voltage, 10 s sample injection time and 500 s separation time, following QIAxcel technology. The QCES automated the process of detecting and measuring the size and quantity of the PCR-amplified DNA products.

Samples positive for the Pan-RT-PCR specific paramyxo-pneumoviruses were then subjected to a Pan-RT-PCR specific for pneumoviruses (PNE-RT-PCR), as described previously with some modifications by setting the annealing temperature at 47 °C [31]. The PCR products were subsequently screened by the QCES platform as mentioned above. The positive PNE-RT-PCR samples were further resolved by 1.5% (w/v) agarose gel electrophoresis and then purified by NucleoSpin® Extract II kit (Macherey-Nagel, Düren, Germany). The purified amplicons were submitted for commercial Sanger sequencing at Macrogen Inc. (Incheon, South Korea).

### Genome sequencing and CPV-specific (PNE)-RT-PCR

Sanger sequencing results of the PNE-RT-PCR positive samples confirmed the presence of CPV in the Thai dog samples. The complete CPV genome sequences were obtained by multiple RT-PCR amplifications using degenerated primer sets designed from multiple alignments of the MPVs, SOV and the various strains of the CPV genomes published in GenBank (Additional file 1: Table S1). The RT-PCRs were performed in a total volume of 50 μL using the Qiagen OneStep RT kit (Qiagen, Hilden, Germany) according to the manufacturer’s recommendations. Briefly, the reactions were comprised of a mixture of QIAGEN OneStep RT-PCR Enzyme Mix, 10 mM of dNTP in 5x QIAGEN OneStep RT-PCR Buffer, 10 μM final concentration of each primer and 5 μL of the template. Thermocycler conditions consisted of 50 °C for 30 s for the RT step, and then amplified by an initial 98 °C for 30 s, followed by 40 cycles of 98 °C for 30 s, 45 °C for 30 s and 72 °C for 1 min, and then a final 72 °C for 7 min. The PCR product(s) was (were) resolved by agarose gel electrophoresis, purified and submitted for Sanger sequencing as above.

### Genome organization and phylogenetic analysis

The derived sequences of individual positive CPV isolates were assembled and then the complete coding genome sequence was constructed using BioEdit v. 7.0.5.3. Because of the close genetic relationship between CPV and MPV, the new CPV TH strains were then compared to the available MPV strains and other CPV strains based on the complete coding genome and, due to the scarcity of complete genome sequences, to the specific G/glycoprotein gene using the MAFFT alignment v. 7 package programs. The deduced amino acids were translated from the complete coding sequences and compared to previous CPV and other related pneumovirus sequences in order to define specific amino acid substitutions of the CPV TH strains. Phylogenetic analysis of the full-length CPV strains and their individual G/glycoprotein gene were constructed using the maximum likelihood (ML), and neighbor-joining (NJ) algorithms with the TN93 + I model for the full-length CPV genomes and the TN93 + G model for the individual G gene the best-fit model of nucleotide substitution according to the Bayesian information criterion and bootstrapping with 1000 replicates were performed. The phylogenetic tree of the new CPV strains was performed using the MEGA 7 software package [32]. Sequence pairwise distances based on the complete genome and the complete G/glycoprotein gene of the newly obtained CPV TH strains were calculated using the maximum Composition Likelihood model in the MEGA 7 software package.

### Genetic recombination

Potential genetic recombination breakpoints of the new CPV TH strains were examined for using the statistically measured methods of RDP, GENECONV, BootScan, MaxChi, Chimaera, SiScan and 3Seq, with the default settings in the Recombination Detection Program (RDP) package version 4.0 [33]. Due to many recombination signals and inconsistent results from different algorithms, only potential breakpoint signals revealed by at least four methods with *p*-values < 0.01 were considered to be a potentially positive recombination [34]. The initial phylogenetic tree constructed with a potential recombinant and its putative major and minor parents were revealed using the RDP 4 package software. The potential recombinant CPV genomes were further subjected to recombination analysis using a similarity plot and bootscan analyses in the SimPlot software package version 3.5.1 [35]. The putative recombinant CPV genome derived from the RDP served as the query in comparison with its parents and modeled with a window size of 200 bp and a step size of 20 bp.

### Selection pressure analysis

Estimation of the substitution rates in the G/glycoprotein gene of the CPVs was achieved by non-neutral selection, which was calculated by the ratio of nonsynonymous (dN) to synonymous (dS) substitutions. The ratio was calculated using the ML phylogenetic reconstruction platform according to the general revisable nucleotide substitution model, available through the Datamonkey web server [36]. To detect non-neutral selection, single likelihood ancestor counting (SLAC) [37], fixed-effects likelihood (FEL) and mixed effects model of evolution (MEME) [38] methods were implemented through the HyPhy software package in the Datamonkey’s sever. Statistical significance at *p* = 0.1 was set with a Bayes factor of 50 for estimation of the dN and dS rates within each codon. The ratio values of dN/dS > 1, dN/dS = 1 and dN/dS < 1 were used to define adaptive molecular evolution (positive selection), neutral mutations and purifying selection (negative selection), respectively.

## Results

### Detection of CPV in clinical samples

Out of 209 dogs, 62 dogs (29.7%) were found to be positive with the PMX-PCR detection. Among these, only six dogs (2.9%) were positive with the PNE-PCR test. The CPV genome was detected in both male and female dogs equally and all six CPV-positive dogs were less than 1 year of age. Regarding their clinical presentation, all CPV-positive dogs revealed inappetence and bronchopneumonia when examined by chest radiograph, but they were all negative for other common CIRDC viruses when tested by multiplex PCRs.

### Genome characterization and organization of the CPV TH strains

**Fig. 1:**
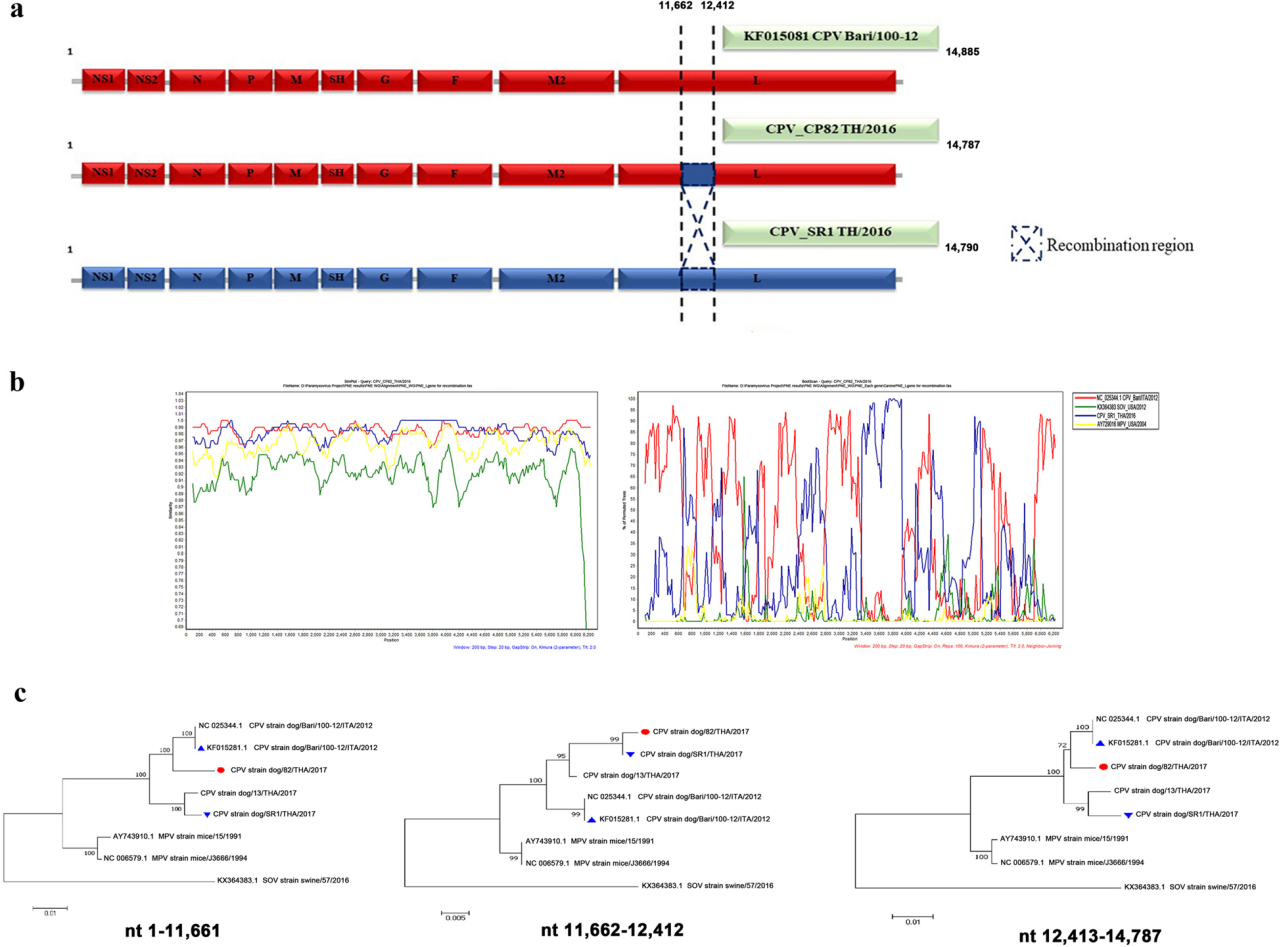
Schematic diagram of the genome organization of the CPV TH strains and the potential recombinant CPV_CP82 TH/2016 strain. **a** The CPV Bari/100–12 (accession no: KF015081; red line) and CPV_SR1 TH/2016 (accession no: MK520878; blue line) strains served as the putative major and minor parents. The recombination event was located at nt 11,662–12,412 in the L gene. **b** Similarity plot and bootscan analysis based on the complete L gene of the recombinant CPV_CP82 TH/2016 as a query. The y-axis indicates the percentage of nucleotide identity and permutated trees for the similarity plot and boot scanning, respectively, within a 200 bp-wide window with a 20-bp step size between plots. **c** The ML phylogenetic trees of the recombinant CPV_CP82 TH/2016 strains (●) and its major (▲) and minor (▼) putative parent strains over three different segments. Bootstrap (1000 replications) values over 50% are shown for each node

Because of the limitation of the amount of the remaining samples, the sequence of the complete coding genome sequence was only completed for three of the PNE-RT-PCR positive samples. These newly identified CPVs in this study were named as CPV_CP13 TH/2015, CPV_CP82 TH/2016 and CPV_SR1 TH/2016, with their genome sequences deposited at GenBank (accession nos. MK520877- MK520879). Nucleotide alignments of all three CPV TH strains (14,787 bp for CPV_CP82 TH/2016 and CPV_SR1 TH/2016 and 14,790 bp for CPV_CP13 TH/2015) were analyzed and revealed 10 consecutive gene sequences (NS1-NS2-N-P-M-SH-G-F-M2-L), as previously described in other CPV strains (Fig. 1a). The NS1 (nt 44–453), NS2 (nt 462–1032) and N (nt 1037–2256) genes encoded 113, 156 and 393 deduced amino acids, respectively, while the P (nt 2268–3174), M (nt 3179–4110) and SH (nt 4113–4508) genes encoded 295, 257 and 92 deduced amino acids, respectively. Furthermore, the main outer membrane proteins were constructed by a chain of 414 and 537 amino acids, encoded by the G/glycoprotein (nt 4511–5844) and F (nt 5861–7523) genes, respectively. The M2 gene (nt 7527–8452), that contained the M2–1 (nt 7583–8113) and M2–2 (nt 8047–8343) parts, encoded 176 and 98 deduced amino acids, respectively, followed by the L gene (nt 8462–14,794) that encoded a huge protein of 2040 amino acids.

**Fig. 2:**
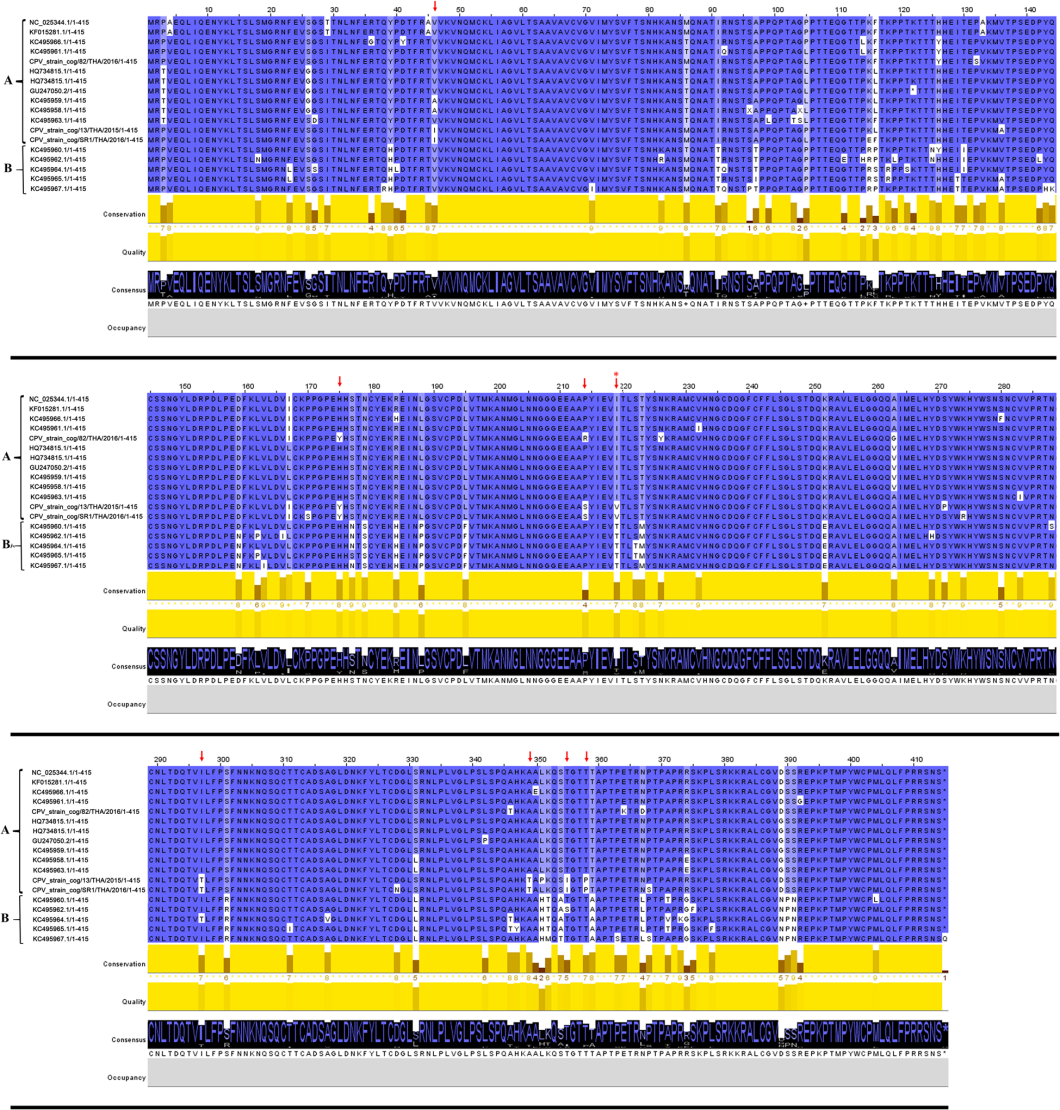
Deduced amino acid alignment of G/glycoprotein comparison between CPV TH and reference strains. Deduced amino acid alignment of CPV TH strains were compared with other reference pneumoviruses, revealing both conserved and variable regions and identifying potentially specific amino acids (↓) for subgroup classification. Both conserved and consensus amino acids are also indicated

The complete coding genome of the three CPV TH strains exhibited the highest nucleotide identity to the Italian and American CPV strains, accounting for 96.4–98.0% and 95.8–96.3%, respectively, whereas they showed a lower identity to the MPVs and SOV at 93.8–94.9% and 87.2–87.9%, respectively. The overall homology of the G/glycoproteins among the CPV strains was high, and the pairwise sequence identities of the CPV TH strains accounted for 90.3–97.6% with the highest identity to the CPV Bari/100–12 strain and the lowest identity to the CPV Texas/110230-11TX strain. Interestingly, analysis of the deduced amino acid sequences of the G/glycoproteins of the CPV TH strains revealed the unique H175Y substitution in all of the CPV TH strains. Among the CPV Thai strains, the CPV_CP82 TH/2016 strain had the different amino acid substitutions V46I, R214S, I219V, I297T, A349T, T355I and T358P (Fig. 2). Other specific amino acid substitutions in the other genes of the CPV TH strains compared to the extant CPVs and closely related pneumoviruses were also encountered, as shown in the Additional file 1: Table S2.

### Phylogeny of the CPV TH strains

**Fig. 3:**
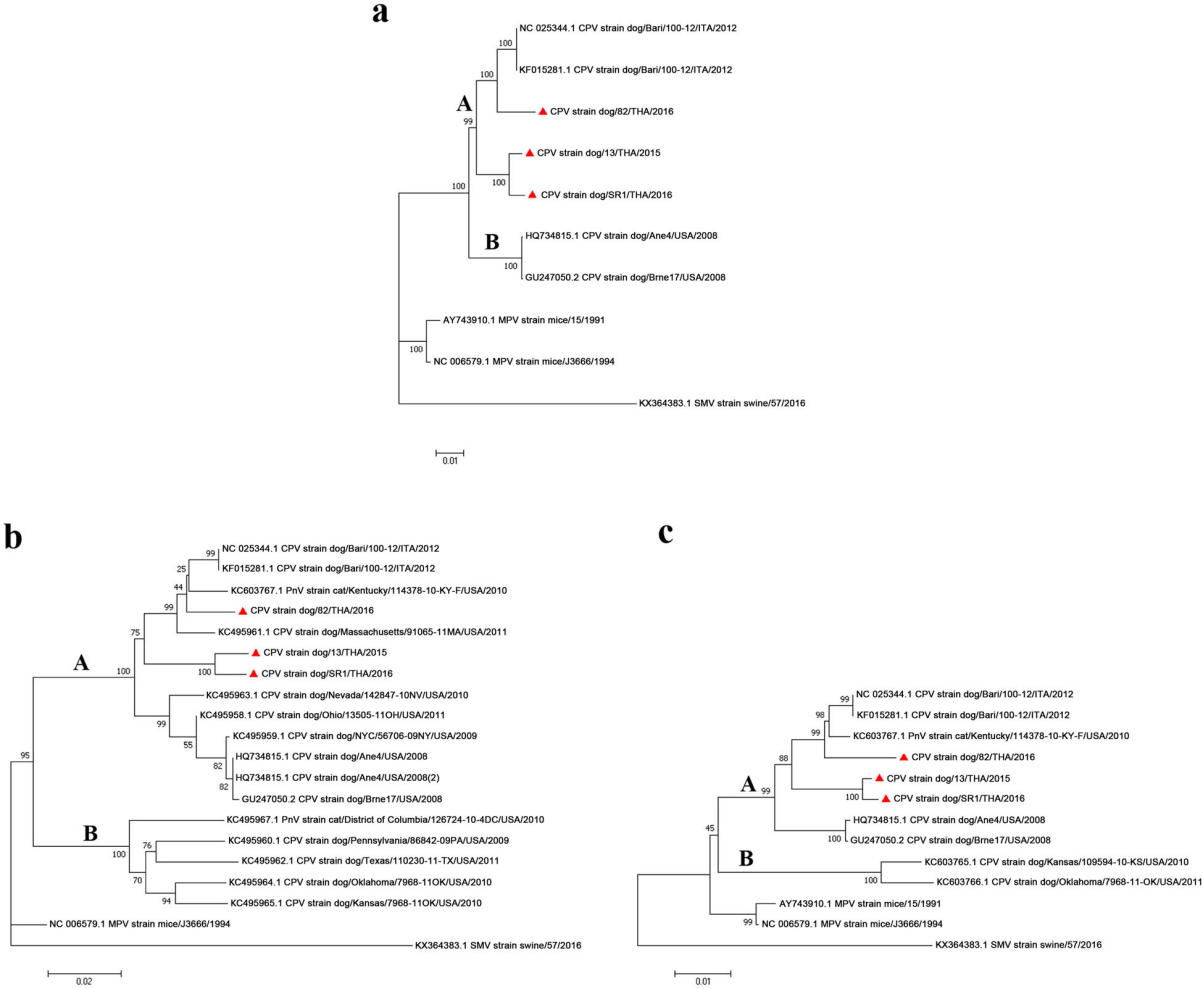
Phylogenetic analysis of CPV strains in Thailand. Phylogenetic trees based on (**a**) the full-length CPV genomes, which revealed that the CPVs were divided into the subgroups of A and B and the CPV strains isolated in Thailand (▲) were clustered with CPV Bari/100–12, and (**b**, **c**) the (**b**) G/glycoprotein and (**c**) F gene, which placed the CPV TH strains in group A of CPVs. The tree topologies of CPVs suggest that the CPV TH strains circulating in Thailand were divergent. Bootstrap (1000 replications) values over 50% are shown for each node

The phylogeny of the CPV TH strains was constructed by alignment of their sequences with the other available complete CPVs and related pneumoviruses, and inferred by the ML character-based and NJ distance-based approaches. These all resulted in trees with similar patterns, where the tree topologies revealed that the CPV TH strains formed two clades, one containing CPV_CP82 TH/2016 that clustered with the CPV Bari/100–12 strain and the other was a cluster of CPV_CP13 TH/2015 and CPV_SR1 TH/2016 that was divergent and formed a novel monophyletic group with Bayesian posterior probability of 1.0 and a ML bootstrap value of 100% (Fig. 3a). In order to evaluate the CPV subtypes, phylogenetic trees based on the G/glycoprotein and F proteins were constructed. These additional phylogenies revealed that all the CPV TH strains belonged to CPV group A and created a new branch with a distinctive cluster of CPV_CP13 TH/2015 and CPV_SR1 TH/2016. However, CPV_CP82 TH/2016 was still clustered with the CPV Bari/100–12 strain (Fig. 3b, c).

### Genetic recombination of the CPV TH strains

In order to further analyze the CPV evolution, we investigated the genetic recombination between strains. Interestingly, CPV_CP82 TH/2016 was identified as a likely recombinant by the RDP software. The potential recombination event was identified and supported by all statistical measurement of recombination algorithms of the RDP, GENECONV, BootScan, MaxChi, SiScan, Chimaera and 3Seq software at a statistical *p*-value of 4.099 × 10^− 5^, 5.200 × 10^− 7^, 7.982 × 10^− 4^, 8.235 × 10^− 3^, 6.214 × 10^− 3^, 7.393 × 10^− 6^ and 5.615 × 10^− 6^, respectively. Among the potential pneumoviruses, the CPV Bari/100–12 and the CPV_SR1 TH/2016 were identified as the likely major (98.7% similarity) and minor (100% similarity) parental strains, respectively.

The putative recombinant CPV_CP82 TH/2016 was then further analyzed for the recombination breakpoint using a similarity plot and bootscan analysis embedded in the SimPlot software package. These methods identified the recombination breakpoint as located at the L gene, similar to the recombination events in the RDP. In the generated similarity plot of the L gene, the nucleotide identity of the CPV_CP82 TH/2016 had a similar high nucleotide substitution to the CPV Bari/100–12 strain at the beginning through the late middle portion of L gene region (red line), while it had obvious nucleotide identities to the CPV_SR1 TH/2016 strain in the nearly middle portion of the L gene (blue line; Fig. 1b). In addition, bootscan analysis confirmed the disparities detected in the phylogenetic topology of the CPV TH strains (Fig. 1b). The recombination event in CPV_CP82 TH/2016 was located at nt 11,662–12,412 in the L gene, which contained sequences derived from the CPV_SR1 TH/2016 and the CPV Bari/100–12 strains, which served as a potential template for the other genes. The recombination event was also confirmed by phylogenetic tree construction of different genome segments (Fig. 1c).

### Selective pressure analysis

The dN and the dS ratio were derived from the G/glycoprotein gene alignment of the various CPV strains. Implementation of SLAC, FEL and MEME supported an overall negative selection pressure for evolution of the G/glycoprotein gene of the CPVs, with a dN/dS ratio < 1. Interestingly, the FEL test showed evidence of potentially positive selection at nt 46, 92, 116, 367 and 374 of the G/glycoprotein gene, whereas the MEME test revealed episodic positive selection at the same sites plus nt 41, 125, 188, 214 and 263 (Additional file 1: Table S3).

## Discussion

The CPV has recently emerged as a pathogenic virus causing respiratory problems in both sheltered and client-owned dogs in France, Greece, Hungary, Italy, Netherlands, Spain, UK [2, 3, 5, 6] and USA [1, 9, 10], whereas another investigation of the pathogen causing respiratory problems in Finland showed no evidence of CPV infection in the dogs with respiratory illness [4]. In this study, we identified the presence of CPV in six (2.9%) CIRDC dogs in Thailand, which is the first evidence of CPV’s emergence in Asia. The CPV incidence in this study is quite lower than other reports. This finding might indicate that the CPV is not the one of common pathogens causing respiratory disease in Thai dog population; however, the present study does not reflect the true epidemiology of the CPV in Thailand. Further large-scale investigations of CPV antigen through its seroprevalence are needed to complete information of CPV prevalence. So far, the CPV-positive dogs in this study revealed severe clinical signs by showing inappetence and bronchopneumonia when examined by chest radiograph; however, the bacterial pathogens associated with the CIRDC were not screened. Thus, the clinical signs relating with CPV-positive dogs in this study need to be interpretation cautiously.

The three characterized CPV TH strains had a high degree of nucleotide identity and were closely related to an Italian strain (Bari/100–12) with an estimated pairwise nucleotide identity of 95.8–96.4% with other CPVs. Based on the criteria of strain classification used in previous studies [39–41], the CPV TH viruses could be considered as novel strains. Thus, we would alternatively name the CPV TH strains as “Asia strains” in this context. Although CPVs have been isolated in Europe and various states of USA, only the complete G/glycoprotein sequences of CPVs isolated in the USA and one full-length genome sequence of the CPV isolated in Italy are currently available, and so this prevents the full-length genetic comparison among CPV prototypes.

Because the G/glycoprotein sequence of many viruses in the *Pneumoviridae* family is variable, divergent and essential for the major neutralizing epitopes of the virus, this provides a basis for molecular studies to define the genetic group, as in HRSV [17, 19, 42, 43]. For the CPVs, previous studies classified this virus into A and B groups, based on analysis of G/glycoprotein gene sequences [9]. In this study, we attempted to compare the specific deduced amino acid profile of the G/glycoprotein from the CPV TH viruses with other CPV strains, which revealed various substitution sites that are specific to the CPV TH strains. However, further genomic analysis with regards to specific deduced amino acid substitutions of other CPV subtypes is needed and essential for the further analysis of CPV isolates.

As sufficient full-length CPV genomes are limited, it was rather hard to compare the genetic diversity through CPV evolution, as this relies on the analysis of the whole genome. Both partial and complete genome analyses were, therefore, conducted in this study. Phylogenetic analysis of the full-length genome and individual G/glycoprotein or F protein genes of the CPV TH strains revealed a similar pattern, presenting a distinct monophyletic cluster of CPV_CP13 TH/2015 and CPV_SR1 TH/2016, while CPV_CP82 TH/2016 was differentially clustered within a new lineage together with the Bari-100/12 strain. These results confirmed that at least two distinct CPV strains were circulating in Thailand.

Through genomic interactions, viruses may extend their virulence by escaping the host immune system, expanding their host range and generating new strains. Genetic recombination is one of the processes that shapes the evolution of many viruses [44, 45], and was recently considered to be forcing genetic diversity in the pneumoviruses [26–28]. Here, we present the first evidence of natural genetic recombination in the CPV_CP82 TH/2016 strain and emphasize that genetic recombination plays a potential role in pneumovirus evolution. Of note, multi-strain infections are one predisposing etiology of natural genetic recombination by the interaction of potential parents in the same host [46–48]. It, therefore, suggested the possibility that at least two strains of the CPV were circulating in Thailand. A large-scale CPV investigation should be further undertaken to establish the level of genetic diversity of the CPVs.

Because viral RNA polymerases lack a proof-reading mechanism, RNA viruses are prone to high mutation rates that allow rapid adaptations to various selection pressures [49, 50]. In this current study, we assessed the selective pressure on the highly variable G/glycoprotein region of the CPVs and identified the codons that appeared to have been under diversifying positive selection as it has been previously found in other pneumoviruses, while most others have been under purifying negative selection, using the FEL and MEME methods, whereas the SLAC test suggested that the G/glycoprotein sequences have only been under negative selection. It is hard to estimate diversifying selection using the alignment of deduced amino acid sequences, because most selective events might be episodic and only occur in some evolutionary clades [27]. The MEME test was used to detect both episodic and pervasive positive selections to reduce the SLAC limitations, as the results indicated [38]. For the HRSVs, the diversifying positive selection sites observing in the G/glycoprotein region were reported and emphasized that these evolutions are more likely impact the host immune response [51]. As positive selection sites were found in the G/glycoprotein sequence in this study, this might imply that the G/glycoprotein of the CPV is a highly mutation region as for viral adaptation to escape the host immunity and might not be as a good target for prevention and intervention strategies. However, result in this study challenged the previous study showing that the G/glycoprotein was under negative pressure without any positive selection sites [9]. These apparently inconsistent results may have been caused by the number of tested sequences and different methods used.

## Conclusions

Our findings indicate that there is a novel lineage of CPV emerging in Thailand and is the first report of CPV in Asia. The Asian CPVs were clearly distinct from previous isolates. Furthermore, we identified a natural genetic recombination in the CPV, suggesting that genetic recombination may shape and force the evolution and diversity of CPV genomes. It is worth noting that the G/glycoprotein evolution is under not only negative selection, but also an episodic/pervasive positive selection that plays a role in shaping the evolutionary process of the CPVs. This finding underlines the need to further investigate the CPV circulating in other countries in Asia and for an analysis of the recombination that might alter the CPV’s pathogenesis and virulence.

## Additional file

Additional file 1: **Table S1.** Primer sets used for CPV complete coding genome sequencing. **Table S2.** Amino acid substitutions of canine pneumovirus Thai strains compared with extant canine, murine and swine pneumoviruses. **Table S3.** Evidence for positive and negative selection using various detection methods. (PDF 199 kb)

## Data Availability

All the data supporting our findings is contained within the manuscript. Three full-length CPV sequences have been deposited in NCBI GenBank under accession numbers MK520877 - MK520879.

## Abbreviations

BRSV: Bovine respiratory syncytial virus
CIRDC: Canine infectious respiratory disease complex
CPV: Canine pneumovirus
dN: nonsynonymous sites
dS: synonymous sites
FEL: Fixed-effects likelihood
HRSV: Human respiratory syncytial virus
MEME: Mixed effects model of evolution
MPV: Murine pneumovirus
PCR: Polymerase chain reaction
PMX: Paramyxoviruses
PNE: Pneumoviruses
RT: Reverse transcription
SLAC: Single likelihood ancestor counting
SOV: Swine orthopneumovirus
TH: Thailand
