# Supplementary material for: Phylogenetic evidence of a novel lineage of canine pneumovirus and a naturally recombinant strain isolated from dogs with respiratory illness in Thailand
Source: BMC Vet Res. 2019 Aug 19;15:300. doi: 10.1186/s12917-019-2035-1 (PMC6700830; doi:10.1186/s12917-019-2035-1)
Supplement: Supplementary file 1 — Table S1. Primer sets used for CPV complete coding genome sequencing. Table S2. Amino acid substitutions of canine pneumovirus Thai strains compared with extant canine, murine and swine pneumoviruses. Table S3. Evidence for positive and negative selection using various detection methods. (PDF 199 kb) [file 12917_2019_2035_MOESM1_ESM.pdf]

Phylogenetic evidence of a novel lineage of canine pneumovirus and a naturally recombinant strain isolated from dogs with respiratory illness in Thailand

Chutchai Piewbang[1], Somporn Techangamsuwan[1, 2, *]

[1]Department of Pathology, Faculty of Veterinary Science, Chulalongkorn University, Bangkok, 10330, Thailand

[2]Diagnosis and Monitoring of Animal Pathogens Research Unit, Faculty of Veterinary Science, Chulalongkorn University, Bangkok, 10330, Thailand.

Supplementary Table S1. Primer sets used for CPV complete coding genome sequencing

Direction	Primer name	Primer sequences (5'-3')
**Forward**	PNE_F12	ATG CAT ACC AAA CCC AC
**Reverse**	PNE_R398	AGC TCA CAT GTG GGG TCA A
**Forward**	PNE_F160	GCW TTC CAY ATA ACC AAC T
**Reverse**	PNE_R1000	TTC TCT GCT GGT TGG RG
**Forward**	PNE_F918	CAT TCY RTA CCC AGG CCA TG
**Reverse**	PNE_R1813	CCC TCA ACY TTG CTG CC
**Forward**	PNE_F1621	GAG GAC TTG ATG CTG T
**Reverse**	PNE_R2471	GAG TTT GAA TGA GCT RCG
**Forward**	PNE_F2244	GTT GAT GAC ACT CCT GAT
**Reverse**	PNE_R3133	GCA TTG CTG GAC ACT ATC T
**Forward**	PNE_F3036	AGA GAT GAG GAA TGC TC
**Reverse**	PNE_R3940	TTC CAG TTC ATA ATC ACA TC
**Forward**	PNE_F3846	GCA TTC AAA CTG CTA AAG
**Reverse**	PNE_R4809	TGR TTT GAT RTG AAW ACA G
**Forward**	PNE_F4746	TCA CAA GTG CTG CTG TG

**Reverse**	PNE_R5511	TCT GCA CAG GTG GTA CA
**Forward**	PNE_F5295	TCC TTT CTG GTT TAA GCA
**Reverse**	PNE_R6291	CAATGCAATCTCACTTTCA
**Forward**	PNE_F6163	TTA TCA TCC AAT GCY TTG
**Reverse**	PNE_R7094	GGC AGA GTC CTT ATT ATA C
**Forward**	PNE_F6964	TGT AAG ATT TCC ACA AGT AA
**Reverse**	PNE_R7906	TCA AGA AAG GTC TAC CA
**Forward**	PNE_F7810	AGG ACT GCT GAG TAT GC
**Reverse**	PNE_R8625	TCA TTG GTA GAC CTT TCT TG
**Forward**	PNE_R9475	TCA TTG GTA GAC CTT TCT TG
**Reverse**	PNE_F8572	TCT AGT CGA GCC TTA TCA ATG
**Forward**	PNE_F9273	GGT CAT CTG CTC AGT AAG T
**Reverse**	PNE_R10252	AAC AGC TTC TCT GCT AA
**Forward**	PNE_F10397	AAG TAG GTG CTC ATT AAT T
**Reverse**	PNE_R11146	GGT CTT TCA AGT CAA CTG AG
**Forward**	PNE_F10105	CAG AGG CTG ACT ATG ATT
**Reverse**	PNE_R11814	ATT CTC GAC CAT CAT GTT
**Forward**	PNE_F11644	CCT GTA TAT CCT CAT GGT
**Reverse**	PNE_R12797	CAA TGA CCA GCA AGA C
**Forward**	PNE_F12494	CTG GRG TGT GTC CTA AT
**Reverse**	PNE_R13187	GTT ACA TTA ACA ACC CAA G
**Forward**	PNE_F13168	CCT TGG GTT GTT AAT G
**Reverse**	PNE_R14013	TTT ATC ATG CTC TGA CT
**Forward**	PNE_F13789	CCT TGT TAT CCT TAC ATA G
**Reverse**	PNE_R14772	ATA CRY TGC TGC TGG
**Forward**	PNE_13900F	TTAA TAG TAT GTG ATG CTG AA
**Reverse**	PNE_14828R	GTT TAC AAC CTG TGA GAT

Supplementary Table S2. Amino acid substitutions of canine pneumovirus Thai strains compared with extant canine, murine and swine pneumoviruses.

Region	Amino acid site	CPV CP13 TH/2015	CPV CP82 TH/2016	CPV SR1 TH/2016	CPV/ Bari/100-12	CPV/ Brne17	CPV/ Ane4	MPV/ J3666	SOV/ 57
**NS1**	36	D	G	G	D	G	G	G	G
**NS2**	12	I	V	I	V	I	I	I	I
	28	G	G	G	G	S	S	G	G
	33	V	V	V	V	M	M	V	V
	67	R	W	R	R	R	R	W	W
	101	S	S	S	A	S	S	S	S
	115	E	E	E	E	K	K	E	E
	120	V	V	M	V	E	E	V	V
	135	V	V	V	V	I	I	I	I
	142	P	P	P	P	L	L	P	P
	156	D	D	D	N	D	D	D	D
**N**	156	C	C	C	Y	C	C	C	C
	161	L	L	L	V	L	L	L	L
	163	V	V	V	G	V	V	I	V
	222	V	V	V	I	V	V	V	V
	342	R	R	R	R	K	K	R	R
	383	N	N	N	N	S	S	N	N
**P**	47	I	M	I	M	I	I	M	M
	59	S	P	S	S	P	P	S	S
	93	T	A	T	A	A	A	V	V
	239	I	I	I	I	V	V	I	I
**M**	45	R	R	R	K	R	R	K	K
	124	N	N	N	D	N	N	N	N
	146	C	C	C	X	C	C	C	C
**SH**	46	I	I	I	V	I	I	I	I
	60	V	V	V	V	A	A	V	I
	77	I	V	V	V	I	I	V	V
	79	A	V	A	V	V	V	V	A
	81	G	G	G	G	S	S	G	G
**G**	26	S	S	S	S	G	G	S	S
	29	I	I	I	T	I	I	I	T
	46	I	V	I	V	V	V	G	V
	105	P	L	L	P	L	L	L	L
	135	A	V	V	V	V	V	A	A
	175	Y	Y	Y	H	H	H	H	Y
	214	S	R	S	P	P	P	P	P
	219	V	I	V	I	I	I	T	I
	297	T	I	T	I	I	I	I	I
	349	T	A	T	T	T	T	T	T
	355	I	T	I	T	T	T	T	T
	358	P	T	P	T	T	T	T	T
**F**	10	F	L	F	L	L	L	L	L
	15	S	N	S	N	N	N	N	N
	60	V	I	V	I	I	I	I	I
	81	S	G	S	S	N	N	S	N
	166	K	E	K	E	K	K	K	K
	473	D	N	D	N	D	D	D	D

	483	G	E	G	E	V	V	E	R
	488	N	N	N	N	T	T	N	N
	507	A	A	A	A	V	V	A	A
	517	L	L	L	L	S	S	L	L
	524	K	K	K	K	R	R	K	K
**M2-1**	57	S	S	S	S	T	T	S	S
	118	A	A	T	A	A	A	T	A
	121	T	T	T	T	V	V	V	V
**M2-2**	82	F	F	F	F	L	L	F	F
**L**	72	S	N	S	N	N/A	N/A	N	N
	76	K	R	K	R	N/A	N/A	R	R
	120	I	V	I	V	N/A	N/A	I	I
	304	T	T	T	I	N/A	N/A	T	T
	327	A	T	A	T	N/A	N/A	A	T
	335	R	K	R	R	N/A	N/A	R	R
	457	E	E	E	K	N/A	N/A	E	E
	705	D	N	D	N	N/A	N/A	N	D
	926	I	V	V	V	N/A	N/A	V	V
	998	R	R	T	R	N/A	N/A	R	R
	1022	S	N	S	N	N/A	N/A	S	S
	1048	G	S	G	S	N/A	N/A	G	G
	1140	I	T	T	I	N/A	N/A	I	I
	1144	K	N	N	K	N/A	N/A	K	K
	1183	T	S	S	T	N/A	N/A	T	T
	1247	L	I	I	L	N/A	N/A	L	L
	1255	N	N	N	S	N/A	N/A	S	S
	1260	K	K	K	R	N/A	N/A	R	R
	1402	S	S	S	T	N/A	N/A	T	T
	1583	K	K	R	K	N/A	N/A	K	K
	1595	A	A	A	T	N/A	N/A	A	A
	1621	R	R	R	K	N/A	N/A	R	R
	1674	K	R	K	K	N/A	N/A	K	K
	1675	R	K	R	K	N/A	N/A	K	K
	1744	G	R	G	G	N/A	N/A	G	G
	1858	D	D	N	D	N/A	N/A	D	D
	1867	K	K	N	K	N/A	N/A	K	K
	1881	F	S	F	S	N/A	N/A	S	S
	1922	S	G	S	G	N/A	N/A	G	G
	2033	V	I	V	I	N/A	N/A	I	I

N/A: No data available

Supplementary Table S3. **Evidence for positive and negative selection using various detection methods**

CPV genome	Selection pressure analysis	SLAC	FEL	MEME
G gene	Positive selection	0	5[a]	10[b]
	Negative selection	20	43	-
	Overall dN/dS	0.378	0.374	0.378

Diversifying selection sites

[a]FEL: 46,92,116,367,374

[b]MEME: 41, 46, 92, 116, 125, 188, 214, 263, 367, 374
